# Supplementary material for: Increase trajectories of tendon micro vibration intensity during ankle plantar flexion: A longitudinal data analysis using latent curve models
Source: PLoS One. 2025 Jul 10;20(7):e0312790. doi: 10.1371/journal.pone.0312790 (PMC12244632; doi:10.1371/journal.pone.0312790)
Supplement: Attachment — Submitted filename: Response to Reviewers.pdf [file pone.0312790.s002.pdf]

Replies to reviewers and editor

Reviewer1

1. Comment 1-1
   - Provide number of time points and intervals at which measurements are taken, using the time metric that best reflects the time of inclusion in the study (typically time from enrollment, or calendar time in studies that involve long enrollment times).
   - Reply 1-1:
     - ✧ Thank you for your suggestion. We would like to address the comment as follows:
       - We agree that in conventional longitudinal studies, it is important to specify the number of time points and the intervals between them using a time metric anchored to study enrollment. In our manuscript, we have explicitly stated the overall data collection period.
       - The present experiment creates repeated-measures data taken within approximately one hour on the same day, rather than longitudinal time-series data. Here the horizontal axis represents stimulus intensity (i.e., exerted muscle strength level). Although the LCM is typically applied to longitudinal data analysis with time as horizontal axis, we believe that applying LCM to such data demonstrates a novel and promising extension of the method's applicability.
2. Comment 1-2
   - Highlight the differences between the time of first measurements and follow-up times.
   - Reply 1-2:
     - ✧ Thank you for your suggestion. We would like to address your comment as follows:
       - As you pointed out, distinguishing between the time of first measurements and follow-up times—especially in terms of participant state—is an important consideration in longitudinal data analysis. In our study, we define the "time of first measurement" as the 0% exerted muscle strength condition, while the "follow-up times" refer to the four subsequent measurements at 25%, 50%, 75%, and 100% exertion.
       - At the time of the first measurement, participants were completely relaxed while fixed to the Cybex device. In contrast, at the follow-up times, they exerted force at predefined levels in the direction of ankle plantar flexion.

Therefore, the primary difference between the measurement points is the presence and level of muscle exertion. All measurements were completed consecutively within approximately one hour. Although this constitutes a repeated-measures design, it does not represent longitudinal time-series data collected over an extended period. Accordingly, we consider it unlikely that any physiological or muscular changes occurred between measurement points. These details are described in the "Exercise protocol" section of the manuscript for your reference.

3. Comment 1-3
   - Describe patterns of missing values across variables at each time point and across time points.
   - Reply 1-3:
     - Thank you for your suggestion. We would like to address your comment as follows:
       - No missing data were present in this study. As shown in Fig. 3, data were reliably collected at each exerted muscle strength level (0%, 25%, 50%, 75%, and 100%) and aggregated into features using equations (1) to (5). For all participants, no missing values occurred for any of the extracted features at the time of first measurement (0%) or at the follow-up times (25%–100%).
4. Comment 1-4
   - Visualize the association between each explanatory variable with the structural variables at baseline.
   - Reply 1-4:
     - Thank you for your suggestion. We would like to address the comment as follows:
       - In our study, we interpret the "baseline" measurement as the data collected at 0% exerted muscle strength. The explanatory variables refer to participant characteristics used in the conditional LCM models (e.g., age, sex, and body size), as shown in Tables 7 and 8. The structural variable at baseline corresponds to the MTG-derived statistic Y_{1j} at 0% exertion, which represents the model intercept.
       - As you rightly noted, visualizing the association between explanatory variables and structural variables at baseline is important for interpreting the results and exploring future applications. However, the main aim of our study was to confirm the existence and amplification pattern of MTG

signals, and our conditional LCM models were focused on the effects of participant characteristics on the slope parameters. Therefore, as shown in Tables 7 and 8, we intentionally did not include the intercept as a function of participant characteristics in the model.

- Nevertheless, we have examined these associations post hoc. For example, the sex-based difference in intercept can be referenced from the α_1 values reported in Tables 3 and 4. Since no explanatory variables showed a prominent or interpretable pattern in relation to the intercept, we chose not to include these results in the main manuscript. Your suggestion provided us with valuable insights into the data, and we sincerely thank you for raising this point.

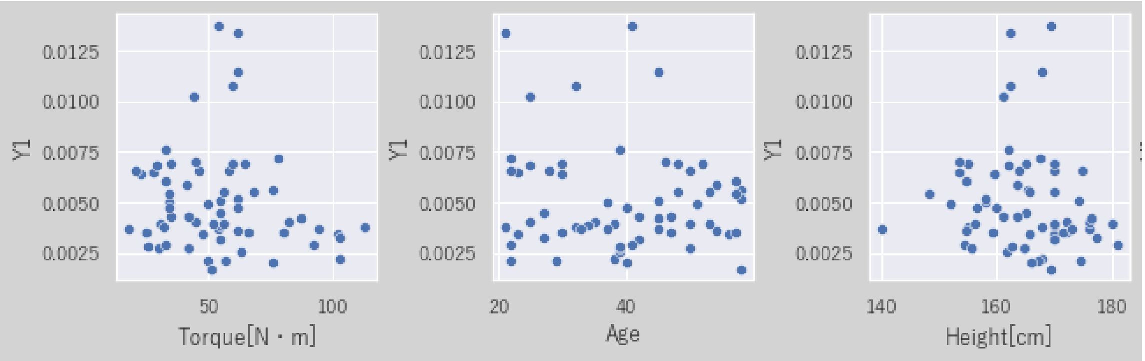

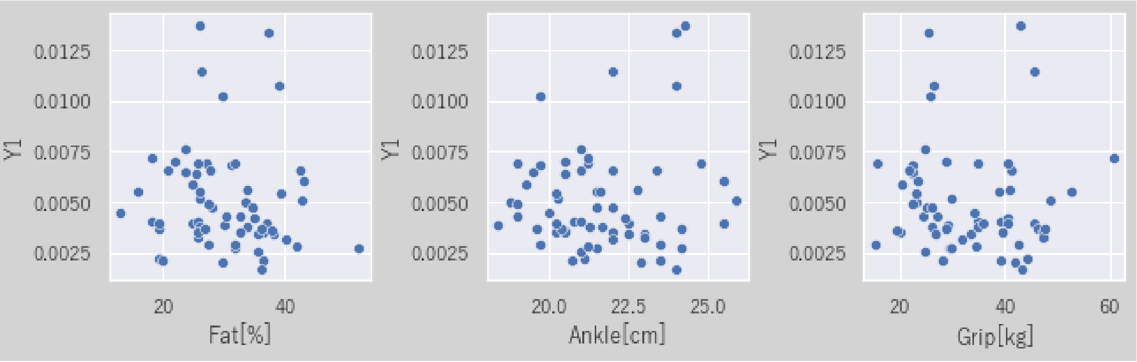

5. Comment 1-5
   - ➢ Summarize changes and variability of variables within subjects.
   - ➢ Reply 1-5:
     - Thank you for your insightful comment. As you correctly pointed out, summarizing within-subject changes and variability is essential when dealing with longitudinal data.
     - To address this, we calculated the range (maximum – minimum) of the absolute values of the sensor data at each exerted muscle strength level for

each participant, and visualized the results using boxplots. As shown, the within-subject variability increases with higher levels of exerted muscle strength. This trend is also reflected in the error variance structure and the θ parameters presented in Tables 3 and 4.

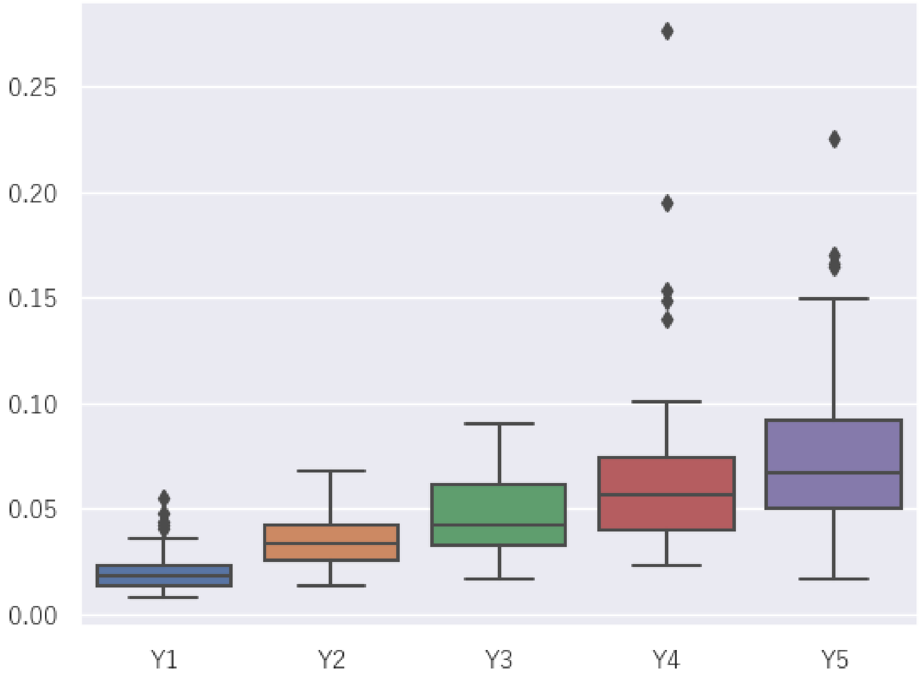

6. Comment 1-6

➢ Describe numerically or graphically the longitudinal trends of the time-varying variables.

➢ Reply 1-6:

✧ Thank you for your suggestion. We would like to address the comment as follows:

- We agree that it is important to describe time-varying variables either numerically or graphically to ensure clarity and interpretability.
- In our study, we recognize that the only time-varying variable is the statistic Y_{ij} derived from the piezoelectric film sensor, which varies according to the level of exerted muscle strength. The longitudinal trend of Y_{ij} is numerically summarized through the parameters of the LCM, as shown in Tables 3 and 4. Additionally, Figures 9 and 10 provide a visual representation of this trend, illustrating both individual-level trajectories and group-level average patterns. We confirm that there are no other time-varying variables in this study apart from Y_{ij}.

Reviewer2

1. Comment 2-1
   - Model Fit Justification: While the piecewise linear LCM fits males well, the borderline fit indices for the quadratic LCM (RMSEA = 0.178) could be discussed further—perhaps exploring alternative nonlinear models.
   - Reply 2-1:
     - ✧ Thank you for your suggestion. We would like to address the comment as follows:
       - We acknowledge that the model fit indices for the quadratic latent curve model (RMSEA = 0.178) fall within a borderline range. From the perspective of practical applications, it would indeed be beneficial to consider alternative nonlinear models that may offer improved fit.
       - However, the primary aim of this study was to explore underlying trends in the biological signal and to deepen our understanding of the observed phenomena. Therefore, we prioritized interpretability in model selection. The piecewise linear model allowed us to visualize how the slope of the MTG signal differs between low and high levels of exerted muscle strength, which provided an important insight. Although the quadratic model yielded a borderline RMSEA, it still demonstrated an acceptable level of fit. This result suggests that it may not be necessary to adopt more complex models that capture highly localized changes. We consider this to be a meaningful finding in itself.
       - That said, we agree with your suggestion that for future applications—especially those aiming at practical prediction or estimation—a better-fitting nonlinear model may be more appropriate. We plan to address this as a future direction of our work.
2. Comment 2-2
   - Data Accessibility: Although ethical restrictions are understandable, providing a de-identified dataset or simulation code (if feasible) would improve reproducibility.
   - Reply 2-2:
     - ✧ Thank you for your insightful comment and valuable suggestion. We have addressed the points as follows:
       - We fully understand the importance of ensuring reproducibility in scientific research, as you rightly pointed out.
       - In response to your suggestion, we consulted with our institutional ethics committee and obtained approval to publicly share an anonymized and

processed version of the dataset. Accordingly, we have deposited the dataset on Figshare in compliance with PLOS ONE's open data policies. The dataset is now publicly accessible at the following DOI: https://doi.org/10.6084/m9.figshare.29123906.v1. We have also updated the Data Availability Statement in the additional information to reflect this change.

3. Comment 2-3
   - Future Work: The hypothesis that MTG reflects muscle fiber contractions could be strengthened by correlating findings with direct physiological measurements (e.g., EMG/MMG) in follow-up studies.
   - Reply 2-3:
     - Thank you for your insightful comments and valuable suggestions. We have addressed the points as follows:
       - As you rightly pointed out, comparing MTG with other physiological signals is essential to better understand its characteristics and limitations. In the present study, we focused on establishing the existence of MTG signals and demonstrating their potential to quantify muscle activity. Therefore, we did not perform comparisons with other signals such as EMG or MMG. However, to determine whether MTG reflects propagated mechanical signals (e.g., MMG), electrical activity (e.g., EMG), or represents an independent signal, simultaneous measurements and comparisons with these modalities are necessary. We recognize this as an important direction for future research.
       - Furthermore, as you suggested, if MTG can complement EMG or MMG in specific contexts, it may be beneficial to combine these methods for practical applications. We sincerely appreciate your suggestion and will consider it in future studies.

Reviewer3

1. Comment 3-1
   - The authors should either conduct more work or provide references that validate that MTG is indeed comparable to EMG or MMG as a reliable physiological measure of muscle activity. It will be important to explain these results in reference to an established benchmark. Or the authors can phrase their concluding comments that their work is an exploratory proxy rather than as a reliable physiological measure for muscle activity.
   - Reply 3-1:
     - ✧ Thank you for your insightful comments and valuable suggestions. We have addressed the points as follows:
       - As you rightly pointed out, interpreting the results of MTG in comparison with established physiological benchmarks (such as EMG or MMG) is essential for validating its reliability as a physiological measure.
       - In this study, we focused on demonstrating the presence of MTG signals and their potential to quantify muscle activity. Therefore, we did not conduct direct comparisons with other physiological signals. To determine whether MTG reflects mechanical activity (e.g., MMG), electrical signals (e.g., EMG), or represents an independent signal, such comparisons are necessary. We consider this a critical direction for future work.
       - Additionally, as you suggested, we do not claim in this paper that MTG is a validated physiological measurement method. Rather, we position MTG as an exploratory proxy with potential relevance. In the Conclusion section, we clearly state that the current study presents a statistical hypothesis, and emphasize the need for follow-up physiological experiments to confirm whether the observed trends correspond to actual physiological processes. We have taken care to avoid overstating the reliability of MTG. We sincerely appreciate your suggestion and will consider it in future studies.
2. Comment 3-2
   - On the minor side, having the figures placed at the end of the work makes it difficult for readers to get proper visual understanding of the work.
   - Reply 3-2:
     - ✧ Thank you for your suggestion. We would like to address the comment as follows:

       We would like to clarify that the placement of all figures at the end of the manuscript is due to the submission formatting requirements of PLOS

ONE. According to the journal's guidelines, figures must be submitted as separate files and cannot be embedded directly in the main manuscript file. However, in the final published version, all figures will be appropriately positioned within the main text. Please note that figure captions are already included in the manuscript and correspond to the relevant text sections for reference.

Reviewer4

1. Comment 4-1
    - The present Data Availability statement does not totally comply with PLOS ONE's open data policies. The authors should consider sharing an unidentified version of the dataset in a public database, or provide a clear explanation if ethical considerations prevents this.
    - Reply 4-1:
        - Thank you for your insightful comments and valuable suggestions. We have addressed the points as follows:
            - As you correctly pointed out, the initial Data Availability Statement did not fully comply with PLOS ONE's open data policy.
            - In response to your suggestion, we consulted with our institutional ethics committee and obtained approval to publicly share an anonymized and processed version of the dataset. Accordingly, we have deposited the dataset on Figshare in compliance with PLOS ONE's open data policies. The dataset is now publicly accessible at the following DOI: https://doi.org/10.6084/m9.figshare.29123906.v1. We have also updated the Data Availability Statement in the additional information to reflect this change.

7. Comment4-2
    - Given that the writing is clear, in my estimation, the introduction and discussion might need a few modifications for simplicity and conciseness.
    - Reply 4-2:
        - Thank you for your valuable comment regarding the simplicity and conciseness of the writing. We would like to address the comment as follows:
            - In the introduction and discussion sections, we aimed to provide detailed explanations to accommodate readers who may not be familiar with exercise physiology, such as statisticians or data scientists. However, as you rightly pointed out, this may have compromised clarity and conciseness. In response to your suggestion, we have revised these sections to improve readability while preserving the intended meaning. We appreciate your feedback and hope the revised version reads more clearly.
