# Supplementary material for: Increase trajectories of tendon micro vibration intensity during ankle plantar flexion: A longitudinal data analysis using latent curve models
Source: PLoS One. 2025 Jul 10;20(7):e0312790. doi: 10.1371/journal.pone.0312790 (PMC12244632; doi:10.1371/journal.pone.0312790)
Supplement: Attachment — Submitted filename: Review_Increase trajectories of tendon.docx [file pone.0312790.s001.docx]

**Increase trajectories of tendon micro vibration intensity during ankle plantar flexion: A longitudinal data analysis using latent curve models**

1. Key Message

The authors present a creative approach where they designed an anklet-type device suitable for the Achilles tendons that allows for the methodological study of muscle activity using mechanotendography (MTG) and latent curve models (LCM).

Results

The authors found that the intensity of the vibration of the tendon exhibited nonlinear increase with exerted muscle strength.

Impression

I feel the authors did a good job with the analysis, as the statistical method of analysis allows for multiple variables to be monitored and accounted for. Also, by having results for both sexes, they are able to show sex-specific differences in muscle activity which is quite insightful. However, I feel that having results that specifically compare to both EMG and MMG would add more strength to their work.

2. What the authors need to improve this study.

The authors should either conduct more work or provide references that validate that MTG is indeed comparable to EMG or MMG as a reliable physiological measure of muscle activity. It will be important to explain these results in reference to an established benchmark. Or the authors can phrase their concluding comments that their work is an exploratory proxy rather than as a reliable physiological measure for muscle activity

On the minor side, having the figures placed at the end of the work makes it difficult for readers to get proper visual understanding of the work.
